# How Can a Multidisciplinary Approach Improve Prognosis of Soft-Tissue Sarcomas of Extremities?

**DOI:** 10.1155/2021/8871557

**Published:** 2021-03-24

**Authors:** Asmae Mazti, Mohamed El Idrissi, Abdelhalim El Ibrahimi, Mustapha El Maaroufi, Ghizlane El Koubaiti, Touria Bouhafa, Samira El Fakir, Samia Arifi, Abdelmajid Mrini, Laila Chbani

**Affiliations:** ^1^Department of Pathology, Hassan II University Hospital, Fez, Morocco; ^2^Department of Traumatology and Orthopedics, Hassan II University Hospital, Fez, Morocco; ^3^Department of Radiology, Hassan II University Hospital, Fez, Morocco; ^4^Medical Center of Biomedical and Translational Research, Hassan II University Hospital, Fez, Morocco; ^5^Department of Radiotherapy, Hassan II University Hospital, Fez, Morocco; ^6^Laboratory of Epidemiology, Faculty of Medicine and Pharmacy, Fez, Morocco; ^7^Department of Oncology, Hassan II University Hospital, Fez, Morocco

## Abstract

Soft-tissue sarcomas are malignant tumors that require good management within specialized centers. Our study aims to assess the benefit of handling these kinds of tumors using the Multidisciplinary Meeting (MDM) approach. The current paper details this approach through a prospective study that has lasted for 42 months in the HASSAN II University Hospital Center, Fez, Morocco. During this research work, 116 cases were selected with an average age of 53 years. In 95.7% of the cases, it was found that the lower limb was the most frequent tumor type (78.4%). Also, ninety-two (92) patients (79.3%) have had a prior biopsy. Ninety-nine (99) patients (85.3%) have received a magnetic resonance imaging scan (MRI) before surgery. Sixty-three (63) patients were operated on, including R0 resection used for 37 patients, R1 used for 21 patients, and R2 used for five patients. As a result, liposarcomas were the most frequent type (30.1%), followed by synovial sarcomas (14.6%), leiomyosarcomas (9.5%), ewing sarcoma (8.6), and undifferentiated pleomorphic sarcomas (7.7%). In addition, neoadjuvant chemotherapy was used for 36 patients. The other 22 patients received adjuvant chemotherapy and/or radiotherapy. The overall survival rate was 60.56 months, which proves a significant improvement, thanks to the multidisciplinary meeting approach. *Conclusion*. The conducted investigation has shown that using MDM for managing soft-tissue sarcomas of extremities improves the patients' survival rate. Moreover, results have proven MDM might allow optimal treatment regarding less local recurrence and metastasis.

## 1. Introduction

Soft-tissue sarcomas are rare, heterogeneous, and vicious. According to their location, the sarcoma tumor could be divided into three categories: the soft-tissue sarcomas of extremities that are the most frequent (60%), the viscera (30%), and the bones (10%) [1].

Note that the treatment of each type can be handled differently. Generally, the treatment of sarcomas depends on early and good prognosis. Practically, surgery can be a solution to soft-tissue sarcomas. This curative treatment relies on surgical resection of the tumor. Such intervention depends on a good functional procedure supported by prior imaging and early biopsy that is absolutely vital.

Therefore, this paper advocates that a multidisciplinary meeting (MDM) should be conducted before any response to a suspected sarcoma. Such a consultation process should involve at least an oncologist, a radiologist, a pathologist, a radiotherapist, and a surgeon. The negligence in carrying out such a meeting would lead to inefficient handling of the tumor and, therefore, ruin any chance of recovery [2, 3].

Accordingly, the current study aims to examine and assess the importance and the impact of such a procedure on the management of soft-tissue sarcomas. Keeping this as an objective, this work investigates 116 cases of soft-tissue sarcomas.

In the second section of this paper, we introduce the methods used for conducting this study, including design, data collection, and statistical analysis. In the third section, we detail the obtained results. Next, in the fourth section, these results are discussed, and the last section summarizes conclusions.

## 2. Methods and Materials

### 2.1. Study Design

Our work is a prospective study that was carried out between 01/01/2017 and 30/06/2020 at the HASSAN II University Hospital in Fez (a tertiary-level hospital). This study is part of a larger research project, which has lasted over 42 months. During the data collection, patients' anonymity and confidentiality were respected. The inclusion and exclusion criteria are listed as follows:

- Inclusion: patients' age ≥18 years
- Patients that are diagnosed with soft-tissue sarcoma of the extremities
- Exclusion: other sarcomas (i.e., bone and viscera)
- Other histological types of cancer

**Figure 1:**
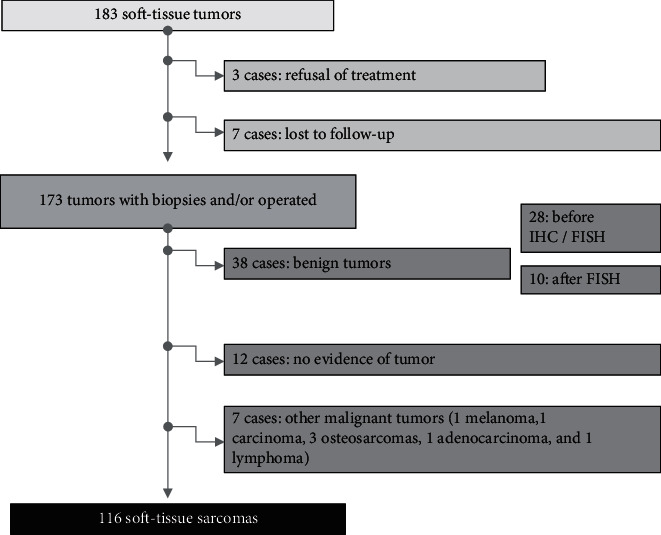
Flowchart of the study.

Consequently, 183 soft-tissue tumors were initially recruited, where 116 cases were diagnosed with a sarcoma tumor. The other 67 were excluded because the pathological diagnosis revealed a benign tumor or a different histological type. Afterward, the patients were subdivided into two groups. Group 1 includes 75 cases whose files are collected from the university hospital. These cases were examined using an MDM procedure before any treatment. Group 2 consists of 41 patients, which involves files from private health instances or patients who received radiological assessments, biopsies, or surgery before being sent to the university hospital (Figure 1).

### 2.2. Data Collection

Data are organized according to three features: (1) the clinical characteristics of patients that include age, gender, sex, history, date of diagnosis, and survival status, (2) the properties of the lesion that involve size, depth, histological type, primary site, and surgical margins, and (3) the structure that initially carries out the management of sarcomas.

The quality of surgical excision (R0, R1, and R2) was assessed as specified by the Union for International Cancer Control (UICC). Therefore, the margin is considered as grossly positive (R2), microscopically positive (R1) (within 1 mm of the inked border), and microscopically negative (R0) (at least 1 mm of normal tissue exists between the tumor and the inked resection margin).

Moreover, our work was conducted in compliance with the international recommendations already available in NCCN and ESMO (4.5). Also, we have compared these recommendations with local practices during the multidisciplinary meetings. In this article, several parameters have been listed and studied. Thus, the impact of soft-tissue sarcoma management can be accurately assessed regarding quality and performance. For example, these parameters include MRI imaging, biopsy, the evaluation of the surgical resection margins regarding the local disease control, and metastatic status.

### 2.3. Statistical Analysis

The collected data were examined and analyzed using the software “SPSS 20.0.” Qualitative variables are expressed using means and medians, whereas quantitative variables are represented using numbers and percentages.

For some criteria, the distribution comparison of qualitative parameters was represented by a chi-squared test, where *p* < 0.05 was considered as being significant. For survival, the method of Kaplan–Meier was adopted. A selected event refers to its first occurrences such as locoregional progression, metastatic progression, and death (all causes are combined). Hence, the original date of the study was the date of diagnosis.

## 3. Results

Of the 116 cases, 71 were males (61.2%) and 45 were females (38.8%). Ages ranged from 18 years to 115 years (age's average was 53.5 years). Most tumors were in the lower limb (78.4%), and the rest were in the upper limb (21.6%). Tumors at deep locations were the most frequent (95.7%), while superficial tumors were less frequent (4.3%). The average size was 12.28 cm (4–32 cm) (Table 1).

Table 2 highlights various indicators. These are used to describe the quality of soft-tissue sarcoma management. Thus, ninety-nine (99) patients (85.3%) have had magnetic resonance imaging (MRI) to evaluate the characteristics of the tumor and to plan a surgical procedure. Ninety-two (92) patients (79.3%) underwent tests and examinations. Most of them (66%) had a prior biopsy, ultrasound, or CT-guided, whereas the other cases (34%) had surgical treatments. Hence, among all the studied cases, 63 were subject to surgical procedures. For 37 patients, the resection was satisfying the (R0) requirement. In 24 cases, it was microscopically positive (R1), while only two patients were presented as grossly positive (R2). Among all the patients who did not have an “in sano: resection R1 or R2,” eight (6.9%) had a surgical operation in our university hospital.

**Figure 2:**
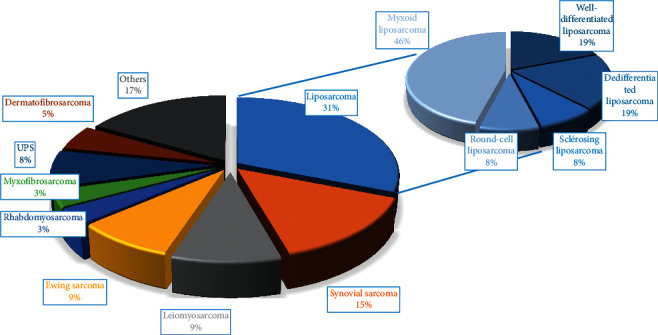
Patients according to histological types.

Figure 2 depicts the histological types in terms of percentages. In this presented series, the most frequent histological diagnoses were liposarcomas (31%). The other ones are established as follows: synovial sarcomas (15%), leiomyosarcomas (9%), Ewing sarcoma (9%), and undifferentiated pleomorphic sarcomas (8%). Accordingly, thirty-six (36) patients have received chemotherapy treatment. Most of them (30 patients) were subject to neoadjuvant chemotherapy (based on the MAI: Adriamycin, isofosfamide, and mesna), EMPTY (vincristine, isofosfamide, doxorubicin, and etoposide), and VAC (vincristine protocols, doxorubicin, and cyclofosfamide). The other six patients were subject to adjuvant chemotherapy. Also, one patient was subject to doxorubicin monotherapy. The other twenty-two (22) patients have benefited from external adjuvant radiation therapy, which was exclusive in nine (9) patients.

**Figure 3:**
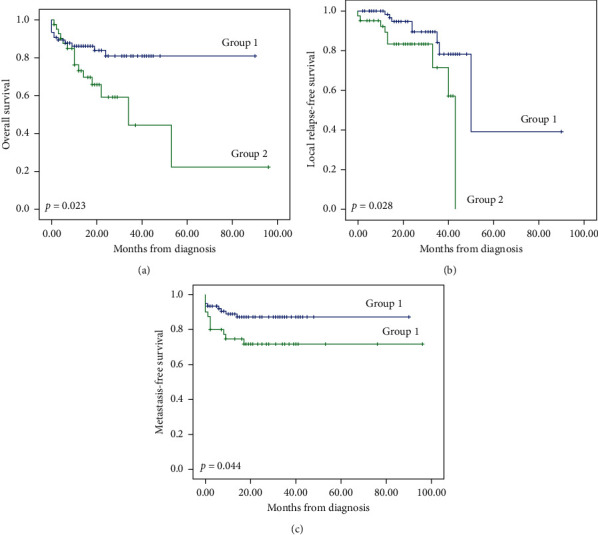
Patient survival of studied groups (overall survival, relapse-free survival, and metastasis-free survival).

As shown in Figure 3, during the evolution course, 23 patients died, and 18 ones have shown local recurrences. Also, the overall survival is 60.561 months.

In Table 3, one can see the performed multivariate analysis along with the parameters of the univariate one. This analysis showed a significant difference between the two groups, especially in terms of the mentioned quality indicators. Compared to the patients treated without being discussed in MDM, results have proven that the patients whose files were discussed during an MDM have benefited from better treatment management and more consistency in clinical practice recommendations. Moreover, for the two groups, the current study has shown that some parameters have no significant impact on patients' treatment (management), such as age, sex, tumor size, tumor location, depth, histologic type, or FNCLCC grade.

## 4. Discussion

Soft-tissue sarcomas are rare and malignant tumors. These are referred to as heterogeneous groups of tumors with a severe prognosis and a banal clinical presentation. Although the handling of sarcomas tumor is well codified through reference systems [4] and recommendations [5], the diagnosis is often a complicated task. Therefore, we advocate multidisciplinary meetings (MDMs) are vital for each stage of the patient's care, including imaging, biopsy, surgery, and adjuvant and neoadjuvant treatments. Such a specific procedure should be conducted mainly within specialized medical structures and requires an oncologist, radiologist, pathologist, radiotherapist, and surgeon.

In such a context, this work endeavors to demonstrate the positive impact of managing tissue sarcomas patients using the MDM approach. These results are specifically related to patients' survival and prognosis. Such findings are indeed supported by previous studies [2, 6–8]. Thus, the cited authors have shown that the overall survival and R0 resection rates were statistically higher when patients are examined within specialized structures. Besides the constant demographic and biological risk factors, other observational studies [9, 10] have noticed that patients' survival is also influenced by how much recommendations and practices are being correctly applied in the management of sarcoma patients. Note that the proposed MDM procedure strives to address different aspects of these parameters.

Regarding the treatment path, the abovementioned recommendations refer to MRI examination and radioguided and surgical biopsy. These combined elements would allow a specific histological diagnosis and successive surgical treatment. In this context, an expert surgeon can easily plan and correctly carry out sarcoma patients.

Typically, the radiologist plays a crucial role, especially in selecting suspicious tumors that require specific management. Accordingly, looking at our cases, one can see that 66 patients (88%) from group 1 and 33 patients (80%) from group 2 underwent a radiological exploration with MRI. These results sound good enough compared to previous publications such as the work of Ray-Coquard et al. [13] (52% of patients) and Haddad et al. [14] (76.5% of patients). In the present study, members of MDM discussed all cases as the patients benefited from coma chest CT in search of distant metastases.

The radiological characteristics that are required during multidisciplinary meetings can be listed as follows [15, 16]:

- Diameter > 50 mm
- Deep localization
- Irregular or lobulated contours
- Presence of irregular and thick intratumoral walls and septa
- Heterogeneity on the T1 and T2 sequences
- Early and prolonged contrast enhancement
- Presence of necrosis

Our study indeed emphasizes the importance of MRI examination before starting patients' treatment. There is indeed a significant difference between the two studied groups as *p* = 0.008. Moreover, we proved that sarcomas patients that have received treatment without prior imaging were the most exposed to a high risk of inappropriate surgery (*p* = 0.028) and local relapse (*p* = 0.001).

After MRI, a biopsy is the first examination to perform in case of a suspected tumor. By comparison with the work of Haddad et al. (72.4%) and Ray-Coquard et al. (42%), in the current work, the biopsy was performed for 68 patients (90.6%) from group 1 and 58.5% from group 2.

Typically, using needles >16G, a surgeon or a radiologist performs a radioguided percutaneous microbiopsy in compliance with the required standards [17]. In this study, 66% of cases (*n* = 61) had a CT-guided biopsy. Such a task should ensure one and definitive surgery for the biopsy pathway and scar. Therefore, the biopsy entrance point should preferably be tattooed. A surgical biopsy might be another option.

Note that surgery is a drastic measure of soft-tissue sarcoma treatment. Thus, it should be performed as a single resection and as an unfragmented specimen. It is noteworthy that this resection should include margins of normal tissue unless there is an anatomical barrier. The quality of the excision is the most important factor of local control [18]. Accordingly, in non-R0 excision, the risk of local recurrence is high [19]. For instance, R1 or marginal excision exposes the patient to a local recurrence risk that could reach 70% [20]. As we stated, MDM is conducted within a specialized instance where, statistically, resections are performed appropriately (R0) [8, 21, 22].

The comparison between the two studied groups (quality of the surgical margins) shows a significant difference in multivariate analysis (*p* = 0.028). Moreover, the risk of local recurrence and distant metastasis decreases in the case of group 1 compared to group 2 (*p* = 0.001 and *p* = 0.034, respectively).

Practically, the current work has shown the importance of MDM regarding overall survival, recurrence-free survival, and metastasis-free survival (*p*, *p* = 0.023, *p* = 0.028, and *p* = 0.044, respectively). Accordingly, the following paragraphs will highlight the context of these results in relation to the data presented by the French Sarcoma Group. Moreover, we will discuss some implications in terms of resources and infrastructure.

The French Sarcoma Group has been known, among others, as a pioneer organization of improvements in the management of sarcoma patients. This organization was established in 2010 by RRePS (reference network in the pathology of sarcomas), NetSarc (clinical reference network), and ResOs (reference network for rare bone sarcomas and rare bone tumors). The organization adopts an approach that revolves around a centralized care system using multidisciplinary discussion and benefiting from expert treatment.

In this context, this French organization has recently conducted a study [23] that involved 12,528 cases, where 9,646 were nonmetastatic and where all patients were followed for 26 months. For patients handled by the multidisciplinary focus group, the local recurrence-free survival was much better. Accordingly, 76.9% of the patients had two-year local relapse-free survival and only 65.4% in the other cases (*p* < 0.001). For both groups, the multivariate analysis of patients included parameters such as sex, age, tumor size, tumor location, histological grade, depth, and handled or not by the multidisciplinary group. The results have shown the handling parameter is a key independent factor that influences relapse-free survival.

Note that the cited work has not assessed the overall survival. In the current paper, we have tried to evaluate this factor. Generally, the obtained results are pretty similar to those presented by the members of the French Sarcoma group. Thus, the overall survival, recurrence-free survival, and metastasis-free survival were significantly higher in the case of patients handled by the multidisciplinary consultation meeting (*p* = 0.023, *p* = 0.028, and *p* = 0.044, respectively).

Also, the quality of the surgery is another factor that determines the quality of care. According to data from the NETSARC network, most patients are operable, and the quality of the resection margins influences relapse-free and overall patient survival. French national indicators show patients operated on by specialized teams from the NetSarc or ResOs networks have a better initial assessment (biopsy and imaging) and a better rate of optimal surgery (R0) and are less subject to revision surgery [24].

In our series, surgery “in sano” (R0) was applied in 58.7% of the cases. The results were statistically significant regarding both groups, in the multivariate analysis (*p* = 0.028) with a lower risk of local recurrence in group 1 compared to group 2 (*p* = 0.001) as well as distant metastases (*p* = 0.034).

Regarding resources and infrastructures, the implications from this study's findings might cover methodological aspects as well as practical ones. Such a procedure would optimize the time and canalize the treatment. It could enhance patients' conditions (i.e., survival rate), and the costs of recovery from inappropriate surgical treatment might decrease.

Typically, the management of soft-tissue sarcoma patients requires specialized centers. These instances can provide the necessary infrastructures and adequate human resources.

For example, a study in Nigeria has shown the impact of the unavailability of relevant radiological tools (i.e., MRI) and the absence of multidisciplinary discussions on sarcomas patients [25]. In such a setting, one would face several challenges to follow or assess survival. In the same context, Adigun et al. [26] have studied STS regarding the pattern, distribution, and issues in a black African community. The cited retrospective study has corroborated the previous results, and it highlighted the difference between the procedures in the Western countries and the African regions (i.e., modern techniques are not commonly available or are usually not affordable).

Ideally, the multidisciplinary meetings should involve an experienced traumatologist, radiologist, pathologist, oncologist, and radiotherapist. Therefore, procedures can be triggered once a suspicious case has risen and before action could be taken. Sometimes (i.e., lack of experience in the nonspecialized centers), advice should be requested from known experts. In complicated or doubtful cases, these human resources are essential for clinical, radiological, histological, or therapeutic aspects.

## 5. Conclusions

In this paper, we detailed our prospective study of soft-tissue sarcomas of the extremities. This work lasted for 42 months, where 116 cases have been studied. The aim was to assess the impact of the multidisciplinary meeting approach on the treatment path of patients. Besides the survival enhancement, MDM helps to achieve optimal treatment with less local recurrence and less metastasis. The collaboration between different specialized members can ensure this successive management of sarcoma patients. Therefore, the management team (MDM) comprises an experienced surgeon, a well-trained radiologist, a pathologist expert, an oncologist who treated many sarcoma patients, and a radiotherapist. This diverse panel of members promoted efficient and accurate handling of suspected sarcoma tumor patients.

## Figures and Tables

**Table 1:** Description of the population.

Characteristics	Total	Group 1	Group 2	*p* value
	*N* = 116	*N* = 75	*N* = 41	
Gender				
Male	71 (61.2%)	46 (39.6%)	25 (21.5%)	
Female	45 (38.8)	30 (25.8%)	15 (13.1%)	0.499
Age at first diagnosis				
Mean (min-max)	53.56 (18–115)	54.81 (18–115)	51.29 (19–85)	
<20	6 (5.2%)	4 (3.4%)	2 (1.7%)	
21–40	31 (26.7%)	18 (15.5%)	13 (11.2%)	
41–60	41 (35.5%)	28 (24.1%)	13 (11.2%)	
61–80	30 (25.9%)	20 (17.2%)	10 (8.6%)	
>80	8 (6.9%)	5 (4.3%)	3 (2.8%)	0.924
Size of the tumor (cm)				
Average	12.28	12.3	11	
Median	10	10	9.5	0.292
Site of the tumor				
Lower limb	91 (78.4%)	59 (50.8%)	32 (27.6%)	
Upper limb	25 (21.6%)	17 (14.6%)	8 (7%)	0.483
Depth				
Deep seated	111 (95.7%)	73 (62.9%)	38 (32.7%)	
Superficial	5 (4.3%)	2 (1.7%)	3 (2.7%)	0.236
Histological subtype (most frequent)				
Liposarcoma	35 (30.1%)	26 (22.4%)	9 (7.7%)	
Leiomyosarcoma	11 (9.5%)	8 (6.9%)	3 (2.7%)	
Ewing sarcoma	10 (8.6%)	7 (6%)	3 (2.7%)	
Synovial sarcoma	17 (14.6%)	11 (9.5%)	6 (5.2%)	
UPS	9 (7.7%)	6 (5.2%)	3 (2.7%)	0.238
Grade (FNCLCC)				
1	15 (12.9%)	8 (6.9%)	7 (6%)	
2	67 (57.7%)	46 (39.6%)	21 (18%)	
3	34 (29.4%)	22 (19%)	12 (10.5%)	0.525
Fluorescence in situ hybridization				
Realized	56 (48%)	42 (36.2%)	14 (12.1%)	
Not realized	60 (52%)	37 (29.3%)	26 (22.4%)	0.030

**Table 2:** Quality criteria for the management of soft-tissue sarcoma.

Parameters	Total	Group 1	Group 2	*p* value
	*N* = 116	*N* = 75	*N* = 41	
MRI before surgery				
Yes	99 (85.3%)	66 (56.9%)	33 (28.4%)	
No	17 (14.7%)	9 (7.7%)	8 (7%)	0.008
Biopsy before surgery				
Yes	92 (79.3%)	68 (58.6%)	24 (20.7%)	
No	24 (20.7%)	8 (7%)	16 (13.7%)	< 0.001
Surgical margins^∗^				
R0	37 (58.7%)	20 (31.7%)	17 (27%)	
R1	21 (33%)	14 (22%)	4 (6.3%)	
R2	5 (8.3%)	2 (3.3%)	3 (4.7%)	0.650
Metastatic status				
M0	89 (76.7%)	62 (53.4%)	27 (23.3%)	
M1	20 (17.2%)	9 (7.7%)	11 (9.5%)	
Mx	7 (6.1%)	5 (4.3%)	2 (1.8%)	0.105
Local relapse				
Yes	18 (15.5%)	8 (6.9%)	10 (8.6%)	
No	98 (84.5%)	67 (57.7%)	31 (26.8%)	0.048

^∗^Data of only operated patients.

**Table 3:** Multivariate analysis.

Parameters	Univariate analysis	Multivariate analysis
Gender	0.499	0.836
Age at the first diagnosis	0.924	0.183
Size of the tumor	0.292	0.198
Site of the tumor	0.483	0.765
Depth	0.236	0.220
Histological subtype	0.238	0.049
Grade	0.525	0.287
Fluorescence in situ hybridization	0.030	0.034
MRI before surgery	0.008	0.022
Biopsy before surgery	<0.001	0.000
Surgical margins	0.650	0.028
Metastatic status	0.105	0.034
Local relapse	0.048	0.001

A Cox model was carried out including all variables in univariate analysis and using a backward selection procedure which entails including all the covariates in the model.

## Data Availability

Data used to support the findings of this study can be obtained from the corresponding author on request.

## Abbreviations

MDM: Multidisciplinary meeting
FISH: Fluorescence in situ hybridization
UPS: Undifferentiated pleomorphic sarcoma
FNCLCC: National Federation of Cancer Campaign Centers
UICC: Union for International Cancer Control
ESMO: European Society for Medical Oncology.
